# Absolute and Relative Reliability of the Timed ‘Up & Go’ Test and ‘30second Chair-Stand’ Test in Hospitalised Patients with Stroke

**DOI:** 10.1371/journal.pone.0165663

**Published:** 2016-10-31

**Authors:** Katrine Lyders Johansen, Rikke Derby Stistrup, Camilla Skibdal Schjøtt, Jacqueline Madsen, Anders Vinther

**Affiliations:** Department of Rehabilitation, Copenhagen University Hospital, Herlev Gentofte Hospital, Herlev, Denmark; University Of São Paulo, BRAZIL

## Abstract

**Objective:**

The timed ‘Up & Go’ test and ‘30second Chair-Stand’ test are simple clinical outcome measures widely used to assess functional performance. The reliability of both tests in hospitalised stroke patients is unknown. The purpose was to investigate the relative and absolute reliability of both tests in patients admitted to an acute stroke unit.

**Methods:**

Sixty-two patients (men, n = 41) attended two test sessions separated by a one hours rest. Intraclass correlation coefficients (ICC_2,1_) were calculated to assess relative reliability. Absolute reliability was expressed as Standard Error of Measurement (with 95% certainty—SEM_95_) and Smallest Real Difference (SRD) and as percentage of their respective means if heteroscedasticity was observed in Bland Altman plots (SEM_95_% and SRD%).

**Results:**

ICC values for interrater reliability were 0.97 and 0.99 for the timed ‘Up & Go’ test and 0.88 and 0.94 for ‘30second Chair-Stand’ test, respectively. ICC values for intrarater reliability were 0.95 and 0.96 for the timed ‘Up & Go’ test and 0.87 and 0.91 for ‘30second Chair-Stand’ test, respectively. Heteroscedasticity was observed in the timed ‘Up & Go’ test. Interrater SEM_95_% ranged from 9.8% to 14.2% with corresponding SRD% of 13.9–20.1%. Intrarater SEM_95_% ranged from 15.8% to 18.7% with corresponding SRD% of 22.3–26.5%. For ‘30second Chair-Stand’ test interrater SEM_95_ ranged between 1.5 and 1.9 repetitions with corresponding SRD of 2 and 3 and intrarater SEM_95_ ranged between 1.8 and 2.0 repetitions with corresponding SRD values of 3.

**Conclusion:**

Excellent reliability was observed for the timed ‘Up & Go’ test and the ‘30second Chair-Stand’ test in hospitalised stroke patients. The thresholds to detect a real change in performance were 18.7% for the timed ‘Up & Go’ test and 2.0 repetitions for the ‘30second Chair-Stand’ in groups of patients and 26.5% and 3 repetitions in individual patients, respectively.

## Introduction

Stroke is one of the most disabling conditions leading to loss of mobility and independency [1]. It is important that functional performance is evaluated with valid and reliable clinical outcome measures. Knowing both the absolute and relative reliability of an outcome measure, enable clinicians and researchers to evaluate the results on a scientific basis and be 95% confident, that a change in the outcome score represents an actual change in performance, and not just a change caused by measurement error and simple test re-test variation.

Walking, standing and sitting on a chair are among the most affected activities for stroke patients and are considered important for the independency of everyday life [2–4]. The timed ‘Up & Go’ test (TUG) and ‘30second Chair-Stand’ test (30s-CST) are both outcome measures widely used in different groups of patients to assess functional performance such as walking, turning and the ability to perform sit to stand-tasks. [4–10]. TUG and 30s-CST are easy to administer compared with other performance measures and can easily be implemented in clinical practice [2, 4, 11, 12].

TUG has been used in several studies to assess functional performance and risk of falling in stroke patients [8, 9, 13, 14]. These are among the most important aspects of stroke rehabilitation, since critical decisions regarding discharge destination, need for further rehabilitation and assistance after discharge depend on assessment of functional performance and risk of falling. However, the reliability of TUG in patients with acute stroke (≤3 months) has never been reported. In chronic stroke patients (≤ 6 months) high relative test-retest reliability with Interclass Correlations Coefficients (ICC) ≥ 0.9 has been observed for TUG [3, 11, 13, 15]. Three of the studies also assessed absolute reliability for TUG and found that the standard error of measurement (SEM) was 1.14 seconds [11], 1.34 seconds [3] and 2.83 seconds [15], respectively.

Different outcome measures are used to assess sit-to-stand tasks [2, 12, 16, 17]. For severely disabled patients it may be too difficult to perform 5 or 10 times sit-to-stand repetitions [2, 12]. Instead of measuring the time it takes to complete a number of repetitions, the 30s-CST measures the numbers of chair-stands a person can perform in 30 seconds, making it possible to assess a wider variation of functional performance levels, with the possible scoring ranging between 0 and ≥20 repetitions depending on the functional capacity of the subjects [12]. The reliability of 30s-CST in patients with acute (≤3 months) or chronic stroke (≥6 months) has not been reported. In patients with dementia [6], patients with osteoarthritis (OA) [18–20] and older adults [21] the test-retest reliability ranged between 0.81–0.98 for 30s-CST. The absolute reliability was 1.26 in patients with dementia [6] and ranged from 0.7 to 1.27 in patients with OA [19, 20] for a group of patients and the absolute reliability for individual patients were 3.49 in patients with dementia [6] and ranged from 1.64 to 2.6 for patients with OA [19, 20]. The purpose was therefore to investigate the relative and absolute reliability of the timed ‘Up & Go’ test (TUG) and ‘30second Chair-Stand’ test (30s-CST) in stroke patients, admitted to an acute stroke unit.

## Materials and Methods

The study was designed as an intra- and interrater intraday reliability study. The reporting of the study follows the Guidelines for Reporting Reliability ad Agreement Studies [22].

### Participants

A convenience sample of hospitalised stroke patients, admitted to the acute stroke unit in the Neurological Department at Herlev Hospital, University of Copenhagen, Denmark, were recruited between October 2013 and June 2014. Patients, who were referred to physical therapy and had a first event of stroke verified by CT/MR-scan or were diagnosed based on clinical symptoms, were screened for eligibility. Patients were not eligible, if they were under the age of 18 years, had a stroke caused by trauma, lacked the ability to sit and stand independently, lacked the ability to walk with or without an assistive device (only relevant for TUG), had pain making testing impossible, or were not able to understand verbal or written information in Danish. Patients were included at any time during their admission when they met the inclusion criteria.

**Fig 1:**
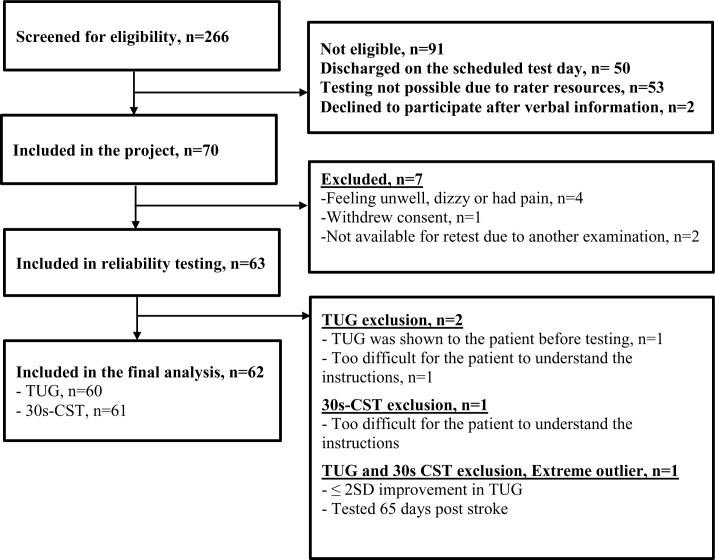
Flow chart of the inclusion of participants.

During the study period 266 patients referred to physical therapy were screened for eligibility by the three physiotherapists participating in the study. These were not consecutive patients as screening resources were not continuously available and consequently more potentially eligible patients were admitted during the period. Of the 266 patients screened, 91 patients (34.2%) were found not eligible and 105 were not included due to early discharge, rater resources and declined participation (Fig 1).

### Procedures

#### Test sessions

The patients attended two test sessions separated by a one hour rest on the same day during hospitalisation. Within each test session both TUG and 30s-CST were performed. The interval between test and re-test was chosen to avoid a change in the functional level of the patients while minimising potential fatigue.

In each test session the patients performed four trials of TUG including a test trial and one trial of 30s-CST with each rater. The patients had a five to ten minute rest before being tested by the second rater. The test leader was present in all the test sessions whereas the raters were not present at each other’s test sessions. The same standardised verbal instructions were given by the raters and the patients were asked if they understood the instruction. If not, the instructions were given again. If the patients did not understand the instructions after three repetitions, the patients were excluded from the study. No talking or encouragement was allowed during testing. To prevent muscular fatigue all patients were brought to the test area in a wheelchair regardless of functional capacity. During the one hour rest between the two test sessions, the patients were instructed to rest either in a chair, wheelchair or in bed and were offered something to drink and eat.

The test sessions took place either in the morning after breakfast or in the afternoon after lunch.

#### Raters

Three experienced physiotherapists participated; two as raters and one as test leader. The raters had two years and three years of experience in the stroke unit, respectively, whereas the test leader had more than 20 years of experience with stroke patients. The test leader collected demographic and clinical data regarding functional capacity prior to the first test session, and recorded the results of each trial of TUG and 30s-CST during the test sessions on a standardised test sheet.

The raters were blinded to each other’s results for the 30s-CST and to the all results of TUG. The raters started and stopped the stopwatch in TUG and the test leader read and recorded the time from the stopwatch, while the raters were kept blinded. To minimize bias the raters changed their measurement sequence between the first and the second test session. The patients performed the tests in the same order in all the test sessions starting with TUG each time.

Both raters received extensive training in the protocol, and a pilot study was conducted prior to the study including 20 hospitalised patients with an acute stroke to calibrate the raters.

#### The New Mobility Score (NMS)

NMS [23] was used to assess pre-stroke functional capacity as an indication of the patient’s independency in everyday life before they had a stroke. This has an impact on the expected outcome on functional capacity after rehabilitation, including an expectation on how the patients will perform in the physical performance measures.

NMS measures the ability to walk; indoors, outdoors and during shopping. A score between 0 and 3 (0: not at all, 1: with help from another person, 2: with an aid, 3: no difficulty) is provided for each function resulting in a total score ranging from 0 (no walking ability) to 9 (fully independent) [23].

The test leader obtained the score by interviewing all the patients.

### Physical performance measures

#### The timed ‘Up & Go’ test

TUG measures the time in seconds it takes an individual to rise from a standard arm chair, walk 3 meters to a line drawn on the floor (at least one foot must touch the line), turn and walk back to the chair to a seated position. No physical assistance is allowed and the use of a walking aid is recorded. The patients wear regular footwear [5].

To achieve a stable performance in TUG, we chose that each patient was given a practice trial followed by three timed trials as recommend by Kristensen et al in patients with hip fracture [24]. Only the best trial was included in the analysis. The following instructions were given to all patients: “On the command “ready, get set, go” please rise from the chair and walk as fast and safely as possible to the line drawn on the floor, turn around and walk back to the chair and sit down. You have to touch the line with at least one foot and you decide which side to turn to. You may use the armrest for arm support to stand up or sit down, if you like”. The rater started the stop watch on “go” and stopped it as soon as the patient was seated again—i.e. when the buttocks reached the seat [24]. The chair had a seat height of 46 cm. Armrest height of 68 cm. The depth of seat was 45 cm and a backrest height of 83 cm.

#### ‘30second Chair-Stand’ test

30s-CST measures the number of sit-to-stand repetitions, with arms crossed over the chest, an individual can do in 30 seconds from a chair with a seat height of 43 cm. The patients were instructed to sit on the chair without touching the backrest and with feet approximately a shoulders width apart and with the knees flexed in 90 degrees [12].

The following instructions were given to all patients: “On the command “ready, get set, go” you have to complete as many sit-to-stands as possible within 30 seconds, and to stand with extended knees, and be fully seated between each stand.”

All patients were tested according to the original manual [12]. If a patient was not able to rise from the chair a modified version of the test was used allowing the patient to use the armrest to rise to a standing position from a chair with a seat height of 46 cm [25]. The modified version was developed by the researchers behind the original 30s-CST and is described in the Senior Fitness Test [25]. When modifying 30s-CST the chair from the TUG was used.

Each patient was given a practice trial with one repetition of sit-to-stand followed by one timed trial. To prevent the chair from moving backwards during testing, it was placed with the backrest against a wall during the 30s-CST.

Both tests were performed according to standardised guidelines [5, 12, 24, 25] and were performed in a long corridor next to the entrance of the Neurological Department at Herlev Hospital.

### Ethics statement

All patients were informed and gave written consent in accordance with the Declaration of Helsinki prior to inclusion in the study. The Research Ethics Committees in the Capital Region of Denmark (j.nr. H-3-2013-FSP10) reviewed the protocol and approved the protocol but found that a formal approval was not required. Danish Data Protection Agency approved the study (j.nr. 2013-41-1601).

### Statistical analysis

It is recommended to have a sample size of at least 50 participants to ensure adequate precision for the estimates of measurement error [11, 26, 27]. The sample size in this study was estimated from this recommendation, and with an expected drop-out of 20%, we planned to include no less than 60 patients during the time of recruitment.

Descriptive statistics and tests for normality (Shapiro-Wilk) were performed for all variables. Results are expressed as mean ± SD and as median and range if the data was not normally distributed. This was, however, only the case for the time from admission to testing data and the NMS data.

Paired t-test was used to assess if significant systematic changes between test trials and between raters were present (p≤0.05).

Intraclass Correlation Coefficient (ICC_2,1_) with corresponding 95% confidence intervals was used to calculate relative reliability. The acceptable ICC_2,1_ was set at a minimal level of >0.8 [28].

Absolute reliability was calculated to establish the variability of repeated measurements using the actual units of the measurements. It was calculated as standard error of measurement (SEM) using a 2-way random ANOVA using the error components to calculate the SEM. The corresponding smallest real difference (SRD) was calculated (SEM x 1.96 x √2) as well as SEM_95_ (SEM x 1.96) to express the variation with 95% certainty for individual subjects and for groups of subjects, respectively.

Bland Altman plots were used to visualise potential systematic variations around the zero line as well as heteroscedasticity. If heteroscedasticity was present SEM% and SRD% were calculated being independent of the units of measurement; SEM% = (SEM/mean)100, SEM_95%_ = (SEM_95_/mean)100 and SRD% = (SRD/mean)100, where mean is the mean of all the TUG and 30s-CST scores respectively from both raters.

Bland Altman plots were also used to identify outliers. An outlier was considered to be present when the difference between the two test sessions was outside 2 standard deviations [11].

All data were analysed using Microsoft Excel program (Microsoft Corp., Redmond, WA, USA), SPSS version 19 (SPSS Inc., Chicago, IL, USA), MedCalc for Windows, Version 14.10.2.0 (MedCalc Software, Ostend, Belgium) and STATA/IC 12.

## Results

Seventy patients were included during the time of recruitment. Seven were excluded due to feeling unwell, withdrawal of consent or were unable to participate in the re-test session. The remaining 63 patients were included in the data analysis (Fig 1). One patient was excluded from the analysis, because the patient was considered to be an extreme outlier due to improvement more than 2SD (18.8 sec) in TUG during the second test session. Moreover, due to an extended length of stay in the department, the patient was tested 65 days post stroke, which was substantially later than the remaining patients (Range: 2–38 days). In the final analysis, 62 patients were included in the study, of whom 61 completed TUG and 62 completed 30s-CST, respectively.

Sixty-two patients aged 71.6 ±13.6 (mean ± SD), range 40–91 years were included in the final analysis of whom 66% were male (n = 41). The time from admission until participation in the test sessions ranged between 2–38 days with a median of 5 days post stroke. NMS pre-stroke ranged between 3 and 9 with a median score of 9. The majority of the patients were thus independent in everyday life prior to admission, even though more dependent patients also were included. Clinical Characteristics are presented in Table 1.

**Table 1:** Clinical characteristics of the participants (n = 62).

	n (%)
TUG	60 (96.8%)
30s-CST	61 (98.4%)
**Type of stroke**	
Ischaemic	54 (87.1%)
Haemorrhagic	6 (9.7%)
Clinically confirmed	2 (3.2%)
**Hemiparetic side**	
Right side	29 (46.8%)
Left side	22 (35.5%)
No paretic symptoms	11 (17.7%)
**Clinically observed problems with balance**^**¶**^	
Yes	54 (87.1%)
No	8 (12.9%)
**Use of assistive device (test day)**	
None	19 (30.6%)
Stick (One or two)	6 (9.7%)
Rollator	37 (59.7%)

^**¶**^Clinically observed problems with balance was defined as observation of balance reactions during activities and locomotion.

Seventy-one percent of the patients performed 30s-CST in accordance with the original test, and 29% performed the test with the standardised modification.

The means and standard deviations for TUG and 30s-CST from the two test sessions are presented in Table 2.

**Table 2:** Summary of the results of TUG (n = 60) and 30s-CST (n = 61).

	Test session 1		Test session 2		
	Mean	SD	Mean	SD	Mean diff ± SD
**TUG (secs)**					
Rater 1	16.2	6.9	15.7	6.8	0.5 ± 2.1
Rater 2	16.1	7.2	15.5	6.8	0.6 ± 2.3
**30s-CST (reps)**					
Rater 1	7.5	2.6	7.8	3.0	0.3 ± 1.5
Rater 2	7.4	2.7	7.6	3.2	0.2 ± 1.3

Secs: Seconds. Reps: Repetitions.

A small but significant learning effect was seen in TUG and 30s-CST (Table 3) when the test results were analysed for the four trials in the order they were actually performed by the patients regardless of the raters, who as previously described changed their testing sequence between test session 1 and test session 2.

**Table 3:** Mean ± SD test results of subsequent test trials of TUG (n = 60) and 30s-CST (n = 61) during the two test sessions (four test trials in total) regardless of raters.

	Testsession 1		Testsession 2	
	Test trial 1	Test trial 2	Test trial 3	Test trial 4
	Mean ± SD	Mean ± SD	Mean ± SD	Mean ± SD
**TUG (secs)**	16.6 ±7.4	15.7 ± 6.8*	15.8 ± 6.9* **	15.4 ± 6.8*
**30s-CST (reps)**	7.1 ± 2.6	7.8 ± 2.8*	7.7 ± 3.0*	7.8 ± 3.2*

Secs: Seconds. Reps: Repetitions.

*Significantly different (p<0.05) from test trial 1.

**Significantly different (p<0.05) from test trial 4.

### Reliability analysis

The results of intrarater and interrater reliability are presented in Table 4 showing high agreement with ICC_2.1_ values ranging from 0.95–0.99 for TUG and from 0.87–0.94 for 30s-CST, respectively.

The smallest measurable difference for a group of patients (SEM_95_) and individual patients (SRD) for TUG and 30s-CST are shown in Table 4. With 95% certainty values above 3.0 seconds for a group of patients and 4.2 seconds for individual patients indicating a real improvement for TUG. More than 2.0 repetitions for a group of patients and 3 repetitions for an individual patient indicated a real improvement for 30s-CST with 95% certainty.

**Table 4:** Relative and absolute reliability for TUG (n = 60) and 30s-CST (n = 61).

	TUG				30s-CST			
	ICC_2,1_	SEM	SEM_95_	SRD	ICC_2,1_	SEM	SEM_95_	SRD
	(95% CI)	(secs)	(secs)	(secs)	(95% CI)	(reps)	(reps)	(reps)
**Intrarater**								
Rater 1	0.96 (0.93–0.97)	1.3	2.5	3.6	0.87 (0.79–0.92)	1.0	2.0	3
Rater 2	0.95 (0.91–0.97)	1.5	3.0	4.2	0.91 (0.85–0.94)	0.91	1.8	3
**Interrater**								
Test session 1	0.97 (0.96–0.98)	1.2	2.3	3.2	0.88 (0.80–0.93)	0.95	1.9	3
Test session 2	0.99 (0.98–0.99)	0.8	1.5	2.1	0.94 (0.90–0.96)	0.75	1.5	2

ICC: Intraclass Correlation Coefficient. SEM: Standard Error of Measurement. SRD: Smallest Real Difference. Secs: Seconds. Reps: Repetitions.

**Fig 2:**
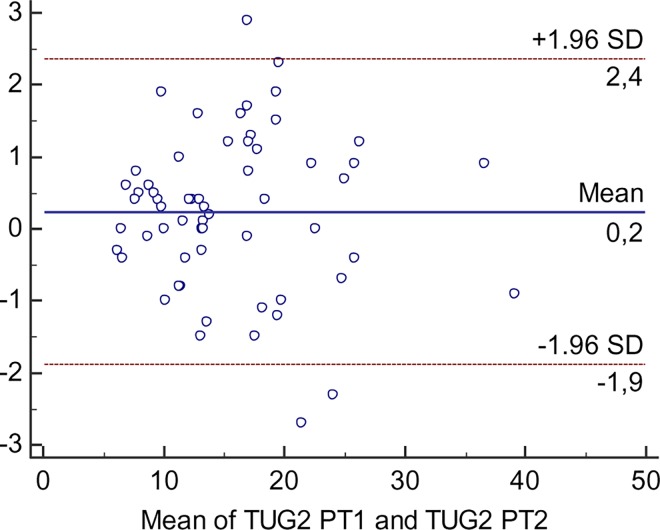
Bland Altman plot of TUG for the interrater reliability.

Heteroscedasticity was observed in the TUG data for both intrarater and interrater with indications of a larger variability for higher test values, as illustrated in the Bland Altman plot (Fig 2). Consequently, the SEM%, SEM_95_% and the SRD% were calculated. Intrarater SEM% ranged from 8.1 to 9.6 and SEM_95_% ranged from 15.8 to 18.7 with corresponding SRD% of 22.3–26.5. Interrater SEM% ranged from 5.0 to 7.3 and SEM_95_% ranged from 9.8 to 14.2 with corresponding SRD% of 13.9–20.1.

## Discussion

To the best of our knowledge, this is the first study to investigate the relative and absolute reliability of TUG and 30s-CST in patients admitted to an acute stroke unit.

The main findings in the present study were 1) excellent relative reliability for both TUG and 30s-CST. 2) The measurement error for TUG and 30s-CST was relatively low, indicating that both outcome measures can be used to detect a real change in functional performance.

To evaluate physical performance after a stroke and changes following an intervention we need reliable outcome measures. It is recommended, that the assessment should include the analysis of agreement between measurements, systematic changes in the mean and measurement error [11, 26]. The statistics applied in the present study are the most commonly used, and the results can be applied in daily clinical practice using the SRD-score for both outcome measures.

The most commonly used method to evaluate reliability is the Intraclass Correlation Coefficient (ICC). There is no clear definition of an acceptable ICC value and often values above 0.70 are used as minimum standards for reporting good reliability [22, 29]. Interpretation of the ICC value should also include considerations for the clinical relevance of the results [22].The ICC values in the present study are considered high and were well above our a priori defined minimum acceptable level of 0.8. Moreover, with one exception the lower limit of the 95% confidence intervals for both TUG and 30s-CST were also above 0.8. Thus the relative reliability observed in the present study seems not only good but also clinical relevant.

### Relative reliability

Few studies have examined the relative reliability of TUG in chronic stroke patients [3, 11, 13, 15] and our results in patients with acute stroke correspond well with these findings. Three studies found ICC values between 0.95 and 0.97 [11,13,15] whereas one study [3] found ICC-values ranging between 0.75–0.84 for the intrarater reliability and between 0.91–0.96 for the interrater reliability.

To our knowledge no studies have investigated the relative reliability of 30s-CST in stroke patients. The high relative reliability in our study was in accordance with those found in patients with mild to moderate dementia (ICC_2,1_ 0.84) [6], in elderly people with cognitive impairments (ICC_3,1_ 0.94) [21], as well as in patients with OA (ICC_2,1_ 0.81) [19] and (ICC_1,1_ 0.95, 0.97, 0.98) [20].

### Absolute reliability

Only three studies have investigated the absolute reliability of TUG in chronic stroke patients showing values of SEM (SEM%) ranging from 1.14 s (8.2%) to 2.83s [3, 11, 15]. The SRD was investigated in two of the studies and the corresponding SRD (SRD%) was 7.84s (28%) [15] and 3.75s (23%) [11], respectively. Similar results were seen in patients with hip fracture with values of SEM (SEM%) of 2.4 s (11%) with corresponding SRD (SRD%) 6.8 s (31%) [30] and in patients with OA with values of SEM 0.84s and major clinically important improvement (MCII) of -1.4s [19].

No studies were found investigating the absolute reliability of 30s-CST in stroke patients. Hesseberg et al. [21] found SEM values of 0.86 repetitions with corresponding values of minimal detectable change (MDC_95_) of 2.4 repetitions in patients with cognitive impairments.Whereas Blakewood et al. [6] found SEM values of 1.26 repetitions and MDC_95_ values of 3.49 in patients with dementia. The results corresponds well with our findings for 30s-CST and similar results are also seen in patients with hip and knee OA with values of SEM of 0.7 repetitions with corresponding MDC_90_ values of 1.64 repetitions [20] and SEM values of 1.27 repetitions with corresponding values of MCII of 2.6 repetitions [19].

### Strengths and limitations

There is a large variation among patients admitted to an acute stroke unit. The varying severity of their hemiparesis and cognitive status makes the group of patients heterogeneous. Out of the 62 patients included in the analysis, 69.4% used an assistive walking device in the test sessions, and the results (Table 2) indicated a large variation in the physical function of the participants. The outlier excluded in this study is an example of the variation in physical and cognitive performance among stroke patients seen in clinical practice. The reason for the extreme improvement in the TUG score during the last test session is unknown.

Since the functional capacity of the patients post stroke was not assessed using standardized measures, the loss of motor function, balance and walking ability as a result of hemiparesis is not transparent. Even though a definition of “clinically observed problems with balance” is given, a more precise description of the functional capacity of the participants in this study would have made comparison to other patient populations more straight forward for clinicians as well as researchers.

To complete TUG the patients must be able to walk a short distance independently with or without an assistive device. 30s-CST requires only the ability to stand up and maintain standing balance. Since most of the patients completed both TUG and 30s-CST the results regarding the reliability of 30s-CST should be interpreted with some caution, when applied to stroke patients without walking ability.

In studies of test-retest reliability it has been recommended, that the sample size should be at least 30, preferably 50 [11, 26, 27]. The larger the sample size, the more reliable the estimates of measurement error. The relatively large sample size of the present study increased the generalisability of the results to stroke patients on other acute stroke units.

In the present study the following was done to minimise bias potentially affecting the test protocol. The test manuals were followed carefully, including standardised verbal instructions and extensive calibration and familiarisation of the two raters. All patients had the same time interval between tests and were tested in the same environment, which was the same location as the physiotherapists normally use for testing.

There is a lack of consensus on how many trials stoke patients have to perform to ensure stable TUG scores. The number of TUG trials performed by stroke patients varies from one to three trials [3, 11, 13, 15], and in the original article participants performed one trial after a familiarisation trial [5]. Faria et al. [3] investigated 16 subjects 1–12.9 years post stroke, and found that the measurement error was very similar between scores of the first trial, the means of two and three trials, and the best and worst values for the three trials.

We chose to use the best of three trials, which has been recommended to ensure stable TUG scores in other hospitalised patients [24]. A small but significant learning effect was, however, still found in the present study–the best of the first three trials were on average approximately one second slower than the best of the second, third and fourth three trials, respectively. A small learning effect in TUG was also seen in chronic patients using the mean of two trials [11]. To minimise the learning effect it could be suggested to perform three trials of TUG followed by a 10 minute pause and then three additional trials of TUG. However, this would be much more time consuming, making TUG less suitable in clinical practice.

A small but significant learning effect was also seen for 30s-CST between the first trial and the second, third and fourth trial, respectively (Table 3). Since the patients in this study exhibited a stabile test score in the second to fourth trial, it could be considered to use the best of two trials of 30s-CST as an alternative to the familiarisation procedure, with just one repetition of sit-to-stand. On the other hand, there is a risk of fatigue depending on the physical condition of the patients.

It is therefore important to consider how to analyse the scores of TUG as well as 30s-CST in future research and in clinical practice, to ensure that the scores reflect a true estimate of the physical performance.

### Implications

TUG and 30s-CST reflect aspects of important and common everyday activities, which make the outcome measures meaningful for the patients. Both outcome measures are suitable to implement in clinical practice, since they are not time consuming, are easy to administer and require no special equipment.

Unlike TUG only few studies have investigated the reliability of 30s-CST for various groups of patients, although it is used to detect improvement of functional performance in many studies. One of the major advantages when using the 30s-CST instead of the 5 or 10 times sit-to-stand test is the possibility of recording a test result, even when the patients are not able to perform a single sit-to-stand repetition, making it possible to test all hospitalised stroke patients on admission and discharge. By applying the results from this study to clinical practice, the physiotherapist can be 95% confident, that an improvement in the test score equal to or more than 3 repetitions indicates a real improvement in functional performance. Moreover, 3 repetitions might also reflect a clinically important difference for the patients. An improvement of 2.6 repetitions was found to be clinically relevant in patients with OA, who were comparable to the present stroke patients regarding 30s-CST performance (≈ 8 repetitions at baseline) [19]. Training sit-to-stand tasks is an essential part of the rehabilitation in stroke patients and implementation of a sit-to-stand test to evaluate functional performance, seems therefore very relevant in clinical practice.

TUG covers a wide range of functional performances since the test requires the strength to stand up from a chair and maintain balance while walking and turning. TUG is found to be a responsive test to detect improvement in mobility during the first three months after stroke with a five seconds improvement in the test score (from 17 to 12 seconds) [31] indicating that TUG can be used in clinical practice to detect a real change in mobility with in the first three months post stroke.

A disadvantage with TUG is the ceiling effect seen in the group of patients with relatively good walking ability [4]. In the study originally describing TUG no assistance beside the participants walking device was permitted [5]. In clinical practice TUG is commonly performed with either minor physical or verbal support from the physiotherapist enabling the physiotherapist to assess functional performance also in the group of patients with lower functional ability. When allowing physical or verbal support, it is important to note that it is a deviation from the original test, and therefore the results of the present study with strict adherence to the original protocol, may not be applied.

Even though both outcome measures are easy to perform two of the 63 participants included in the study still had difficulty understanding the verbal instructions. This could indicate that hospitalised patients with stroke also need a visual instruction. When implementing the outcome measures, it is important to calibrate the raters, to ensure that they give the same verbal instructions regarding the actual wording, the tone and the gesticulation.

## Conclusion

The timed ‘Up & Go’ test and ‘30second Chair-Stand’ Test showed excellent reliability in hospitalised patients with a first event of stroke. Based on the present results we recommend that the threshold used to detect a real change for a group of patients is 18.7% for TUG, and 2.0 repetitions for 30s-CST in research settings. For individual patients in the clinical setting a change of 26.5% for TUG and 3 repetitions for 30s-CST are recommended to be interpreted as a real change in performance.

## Supporting Information

S1 Dataset (XLS)

## References

[1] Ng S. Balance ability, not muscle strength and exercise endurance, determines the performance of hemiparetic subjects on the timed-sit-to-stand test. Am J Phys Med Rehabil. 2010;89(6): 497–504. 10.1097/PHM.0b013e3181d3e90a 20216059

[2] Silva PFS, Quintino LF, Franco J, Faria CDCM. Measurement properties and feasibility of clinical tests to assess sit-to-stand/stand-to-sit tasks in subjects with neurological disease: a systematic review. Braz J Phys Ther. 2014;18(2): 99–110. 10.1590/S1413-35552012005000155 24839043 PMC4183244

[3] Faria CDCM, Teixeira-Salmela LF, Gomes Neto M, Rodrigues-de-Paula F. Performance-based tests in subjects with stroke: outcome scores, reliability and measurement errors. Clinical Rehabilitation. 2011;26(5): 460–9. 10.1177/0269215511423849 22008883

[4] Hafsteinsdottir TB, Rensink M, Schuurmans M. Clinimetric properties of the Timed Up and Go Test for patients with stroke: a systematic review. Top Stroke Rehabil. 2014;21(3):1 97–210. 24985387 10.1310/tsr2103-197

[5] Podsiadlo D, Richardson S. The timed "Up & Go": a test of basic functional mobility for frail elderly persons. J Am Geriatr Soc. 1991;39(2): 142–8. 1991946 10.1111/j.1532-5415.1991.tb01616.x

[6] Blankevoort CG, van Heuvelen MJ, Scherder EJ. Reliability of six physical performance tests in older people with dementia. Phys Ther. 2013;93(1): 69–78. 10.2522/ptj.20110164 22976448

[7] Holm B, Tange Kristensen M, Husted H, Kehlet H, Bandholm T. Thigh and Knee Circumference, Knee-Extension Strength, and Functional Performance After Fast-Track Total Hip Arthroplasty. American Academy of Physical Medicine and Rehabilitation. 2011;3: 117–24. 10.1016/j.pmrj.2010.10.019 21333950

[8] Faria CDCM, Teixeira-Salmela LF, Nadeau S. Predicting levels of basic functional mobility, as assessed by the Timed “Up and Go” test, for individuals with stroke: discriminant analyses. Disability and Rehabilitation. 2012:Early Online: 1–7. 10.3109/09638288.2012.690497 22671699

[9] Paquet N, Desrosiers J, Demers L, Robichaud L, BRAD. Predictors of daily mobility skills 6 months post-discharge from acute care or rehabilitation in older adults with stroke living at home Disability and Rehabilitation. 2009;31(15): 1267–74. 10.1080/09638280802621374 19294546

[10] Jorgensen MG, Laessoe U, Hendriksen C, Nielsen OBF, Aagaard P. Efficacy of Nintendo Wii Training on Mechanical Leg Muscle Function and Postural Balance in Community-Dwelling Older Adults: A Randomized Controlled Trial. J Gerontol A Biol Sci Med Sci. 2012:1–8. 10.1093/gerona/gls222 23114461

[11] Flansbjer U-B, Holmbäck AM, Downham D, Patten C, Lexell J. Reliability og gait perfomance tests in men and women with hemiparesis after stroke. J Rehabil Med. 2005;37: 75–82. 10.1080/16501970410017215 15788341

[12] Jones C, Rikli R, Beam W. A 30-s chair-stand test as a measure of lower body strength in community-residing older adults. Res Q Exerc Sport. 1999;70: 113–9. 10.1080/02701367.1999.10608028 10380242

[13] Ng SS, Hui-Chan CW. The Tiimed Up & Go Test: Its Reliability and Association With Lower-Limb Impairments and Locomotor Capacities in People With Chronic Stroke. Arch Phys Med Rehabil. 2005;86: 1641–7. 10.1016/j.apmr.2005.01.011 16084820

[14] Alexandre TS, Meira DM, Rico NC, Mizuta SK. Accuracy of Timed Up and Go Test for screening risk of falls among community-dwelling elderly. Rev Bras Fisioter. 2012;16(5): 381–8. 22858735 10.1590/s1413-35552012005000041

[15] Hiengkaew V, Jitaree K, Chaiyawat P. Minimal Detectable Changes of the Berg Balance Scale, Fugl-Meyer Assessment Scale, Timed “Up & Go” Test, Gait Speeds, and 2-Minute Walk Test in Individuals With Chronic Stroke With Different Degrees of Ankle Plantarflexor Tone. Arch Phys Med Rehabil. 2012;93: 1201–08. 10.1016/j.apmr.2012.01.014 22502805

[16] Guralnik J, Simonsick E, Ferrucci L, Glynn R, Berkman L, Blazer D, et al A short physical performance battery asessing lower extremity function: association with self-reproted disability and prediction of mortality and nursing home admission. J Geron Med Sci. 1994;49: 85–94. 10.1093/geronj/49.2.m85 8126356

[17] Csuka M, McCarty D. Simple method for measurement of lower extremity muscle strength. Am J Med. 1985;78:7 7–81. 10.1016/0002-9343(85)90465-6 3966492

[18] Dobson F, Hinman RS, Hall M, Terwee CB, Roos EM, Bennell KL. Measurement properties of performance-based measures to assess physical function in hip and knee osteoarthritis: a systematic review. Osteoarthritis Cartilage. 2012;20(12):1 548–62. 22944525 10.1016/j.joca.2012.08.015

[19] Wright AA, Cook CE, Baxter DG, Dockerty JD, Haxby Abbott J. A Comparison of 3 Methodological Approaches to Defining Major Clinically Important Improvement of 4 Performance Measures in Patients With Hip Osteoarthritis. Journal of orthopaedic & sports physical therapy. 2011;41(5): 319–27. 21335930 10.2519/jospt.2011.3515

[20] Gill S, McBurney H. Reliability of performance-based measures in people awaiting joint replacement surgery of the hip or knee. Physiother Res Int 2008;13(3): 141–52 10.1002/pri.411 18697226

[21] Hesseberg K, Bentzen H, Bergland A. Reliability of the Senior Fitness Test in Community-dwelling Older People with Cognitive Impairment. Physiother Res Int. 2015;20(1): 37–44. 10.1002/pri.1594 24925585

[22] Kottner J, Audigé L, Brorson S, Donner A, Gajewski BJ, Hróbjartsson A, et al Guidelines for Reporting Reliability and Agreement Studies (GRRAS) were proposed. Journal of Clinical Epidemiology. 2011;64:96–106. 10.1016/j.jclinepi.2010.03.002 21130355

[23] Kristensen MT, Bandhold T, Foss NB, Ekdahl C, Kehlet H. High inter-tester reliability if the new mobility score in patients with hip fracture. J Rehabil Med. 2002;40: 589–91. 10.2340/16501977-0217 18758678

[24] Kristensen MT, Ekdahl C, Kehlet H, Bandhold T. How Many Trials Are Needed to Achieve Performance Stability of the Timed Up & Go Test in Patients With Hip Fracture? Arch Phys Med Rehabil 2010;91: 885–9. 10.1016/j.apmr.2010.01.021 20510979

[25] Rikli RE, Jones J. Senior Fitness Test Manual. IL: Human Kinetics Champaign; 2001.

[26] Hopkins W. Measures of reliability in sports medicine and science. Sports Med. 2000;30:1–15. 10907753 10.2165/00007256-200030010-00001

[27] Walter S, Eliasziw M, Donner A. Sample size and optimal designs for reliability studies. Statistics in medicin. 1998;17: 101–10. 10.1002/(sici)1097-0258(19980115)17:1<101::aid-sim727>3.0.co;2-e 9463853

[28] Shrout PE. Measurement reliability and agreement in psychiatry. Statistical Methods in Medical Research. 1998;7: 301–17. 9803527 10.1177/096228029800700306

[29] Portney LG, Watkins MP. Foundations of clinical research—applications to practice 3rd Edition ed. Upper Saddle River: Pearson Education Inc.; 2009.

[30] Kristensen MT, Henriksen S, Stie SB, Bandholm T. Relative and absolute intertester reliability of the timed up and go test to quantify functional mobility in patients with hip fracture. J Am Geriatr Soc. 2011;59(3): 565–7. 10.1111/j.1532-5415.2010.03293.x 21391955

[31] Persson CU, Danielsson A, Sunnerhagen KS, Grimby-Ekman A, Hansson P. Timed Up & Go as a measure for longitudinal change in mobility after stroke–Postural Stroke Study in Gothenburg (POSTGOT). J Neuroeng Rehabil. 2014; 11:83 10.1186/1743-0003-11-83 24885868 PMC4037114

